# Development and validation of a prediction model for mechanical ventilation based on comorbidities in hospitalized patients with COVID-19

**DOI:** 10.3389/fpubh.2023.1227935

**Published:** 2023-07-14

**Authors:** Yi Zhang, Yang-Jie Zhu, Dao-Jun Zhu, Bo-Yang Yu, Tong-Tong Liu, Lu-Yao Wang, Lu-Lu Zhang

**Affiliations:** ^1^Department of Gastroenterology, Changzheng Hospital, Naval Medical University, Shanghai, China; ^2^Department of Gastroenterology, The Affiliated Hospital of Southwest Medical University, Luzhou, China; ^3^Department of Military Health Management, College of Health Service, Naval Medical University, Shanghai, China; ^4^Operating Room, West China Hospital, Sichuan University, Chengdu, China; ^5^West China School of Nursing, Sichuan University, Chengdu, China

**Keywords:** COVID-19, comorbidity, hospitalization, mechanical ventilation, prediction model, retrospective study

## Abstract

**Background:**

Timely recognition of respiratory failure and the need for mechanical ventilation is crucial in managing patients with coronavirus disease 2019 (COVID-19) and reducing hospital mortality rate. A risk stratification tool could assist to avoid clinical deterioration of patients with COVID-19 and optimize allocation of scarce resources. Therefore, we aimed to develop a prediction model for early identification of patients with COVID-19 who may require mechanical ventilation.

**Methods:**

We included patients with COVID-19 hospitalized in United States. Demographic and clinical data were extracted from the records of the Healthcare Cost and Utilization Project State Inpatient Database in 2020. Model construction involved the use of the least absolute shrinkage and selection operator and multivariable logistic regression. The model’s performance was evaluated based on discrimination, calibration, and clinical utility.

**Results:**

The training set comprised 73,957 patients (5,971 requiring mechanical ventilation), whereas the validation set included 10,428 (887 requiring mechanical ventilation). The prediction model incorporating age, sex, and 11 other comorbidities (deficiency anemias, congestive heart failure, coagulopathy, dementia, diabetes with chronic complications, complicated hypertension, neurological disorders unaffecting movement, obesity, pulmonary circulation disease, severe renal failure, and weight loss) demonstrated moderate discrimination (area under the curve, 0.715; 95% confidence interval, 0.709–0.722), good calibration (Brier score = 0.070, slope = 1, intercept = 0) and a clinical net benefit with a threshold probability ranged from 2 to 34% in the training set. Similar model’s performances were observed in the validation set.

**Conclusion:**

A robust prognostic model utilizing readily available predictors at hospital admission was developed for the early identification of patients with COVID-19 who may require mechanical ventilation. Application of this model could support clinical decision-making to optimize patient management and resource allocation.

## Introduction

Coronavirus disease 2019 (COVID-19), caused by the novel severe acute respiratory syndrome coronavirus 2, is associated with a significantly high mortality rate in patients who progress to respiratory failure (1). Approximately 14–33% patients with COVID-19 progress to respiratory failure and require mechanical ventilation (2–4). The escalating COVID-19 cases poses enormous challenges for healthcare systems and strain the availability of mechanical ventilation. Delayed recognition of respiratory failure and requirement of mechanical ventilation can increase the risk of hospital mortality (5). Therefore, the development of risk stratification tool that enables early identification of patients with COVID-19 who may require mechanical ventilation is essential to optimize resource allocation and prevent clinical deterioration.

Although various risk stratification models have been developed to identify patients with high risk of severe outcomes (6–9), most of these models are at high risk of bias (10). Furthermore, these models have major limitation, such as inadequate sample sizes and inappropriate model evaluation, which could lead to model overfitting and optimistic model performance (11). Moreover, many prediction models have incorporated abnormal imaging manifestations and some certain biochemical results as predictor variables due to their significant association with mechanical ventilation (3, 12). However, these parameters were frequently unavailable at the time of hospital admission, which consequently impacts the clinical utility of the model for early identification of high-risk patients. Hence, it is imperative to develop a prediction model that addresses these concerns by employing an adequate sample size, appropriate evaluation techniques, and incorporating predictor variables that are routinely recorded upon hospital admission.

Previous studies have confirmed the association between demographic characteristics, comorbidities (such as diabetes, renal disease, and neurologic disorders), and the necessity for mechanical ventilation in patients with COVID-19 (12–14). Risk stratification models based on variables that are readily available at hospital admission hold the potential to serve as invaluable tools for facilitating clinical triage, judicious allocation of limited resources, and reduce hospital mortality, particularly for those with rapid progression of critical illness.

The primary objective of this study was to develop and validate a prediction model for patients with COVID-19, aimed at accurately identifying those individuals who would ultimately require mechanical ventilation, using demographic characteristics and comorbidity variables as key predictors.

## Methods

### Study design and participants

This retrospective study included patients admitted with COVID-19 utilizing the Healthcare Cost and Utilization Project (HCUP) State Inpatients Database (SID) of United States (US) in 2020, which contains the universe of the State’s hospital inpatient discharge records. All the data users adhered to a Data Use Agreement, and the need for informed consent was waived due to de-identification of individual information. The Ethics Committee of the Naval Medical University approved this study (No. 2021LL024). Model development, validation and reporting were conducted in adherence with the guidelines of the Transparent Reporting of a Multivariable Prediction Model for Individual Prediction or Diagnosis (15).

Patients hospitalized with an admitting diagnosis of COVID-19 were included in this study. COVID-19 hospitalization cases were identified based on the International Classification of Disease, 10th revision, Clinical Modification (ICD-10-CM) code U071 (16). Patients were excluded if they were aged <18 years or had a length of stay (LOS) < 2 days. Records with missing values were excluded, as only six missing data were observed in 94,631 patients.

### Outcomes

The primary outcome was the need for mechanical ventilation support, which was identified based on the ICD-10 Procedure Coding System (PCS) codes 5A1935Z, 5A1945Z, and 5A1955Z (17).

### Predictor variables

Age, sex, and Elixhauser Comorbidity Index (ECI) were selected as potential predictor variables due to their significant association with clinical outcomes of patients with COVID-19, as established in previous studies (3, 14). The ECI, encompassing 38 binary comorbidity variables, has been demonstrated to significantly impact mortality rates and resource allocation within the hospital setting (18). For the purpose of analysis, age was categorized into four distinct group: < 60, 60–69, 70–79, or ≥ 80 years, to simplify calculation and interpretation.

### Model development

Patients included in the HCUP SID dataset from Florida in 2020 were allocated to the training set and used for developing the model. Adhering to the principle of at least 10 events per candidate predictor parameter, a total of 5,971 outcome events in the training set was sufficient for developing robust models (19).

To address potential issues of overfitting and collinearity among variables, feature selection was performed using the least absolute shrinkage and selection operator (LASSO) technique, incorporating a 10-fold cross-validation approach (20). The selection of the optimal lambda value for the LASSO regression, which was used to fit the prediction model, followed the one standard error rule. Predictor variables identified through LASSO regression were further evaluated using multivariable logistic regression employing the Enter method. A nomogram for predicting the need of mechanical ventilation support in patients with COVID-19 was constructed based on the results of multivariable logistic regression.

The area under a receiver operating characteristic (ROC) curve was used to assess the discrimination of the model. The optimal cut-off point was determined by identifying the threshold that maximized the Youden index. The agreement between the predicted and observed applications of mechanical ventilation was assessed using a calibration curve. Additionally, decision curve analysis (DCA) was performed to compare the clinical utility of the nomogram and the default strategies of “treat all” or “treat none” by calculating the net benefits at different threshold probabilities.

### Model validation

To validate the prediction model, patients included in the HCUP SID of Kentucky in 2020 were allocated to the validation set. The discrimination, calibration, and clinical utility of the prediction model were evaluated by ROC analysis, the calibration curve, and DCA, respectively.

### Sensitivity analysis

Sensitivity analyses were conducted to evaluate the discriminatory performance of the prediction model under different scenarios. If dataset included patients aged <18 years or those with an LOS < 2 days, a sensitivity analysis was conducted using the complete data. Furthermore, considering the existing evidence suggesting variability in the risk of mechanical ventilation among patients with COVID-19 across different ethnicities (12), additional sensitivity analysis was performed to examine model’s performance within various ethnic groups.

### Statistical analysis

Continuous variables were presented as either mean (standard deviation) or median (interquartile range, IQR), whereas categorical variables were expressed as percentages. The Kruskal–Wallis test, Chi-square test, or Fisher’s exact test were used to compare the demographic and clinical characteristics of patients who required mechanical ventilation and those who did not, as appropriate. Multivariable logistic regression analyses were conducted, and the results were reported as coefficients and odds ratios (OR) with corresponding 95% confidence intervals (CI). Results were considered statistically significant for *p* < 0.05. R software (version 4.3.0) was used to perform all the statistical analyses.

## Results

### Baseline characteristics

**Figure 1:**
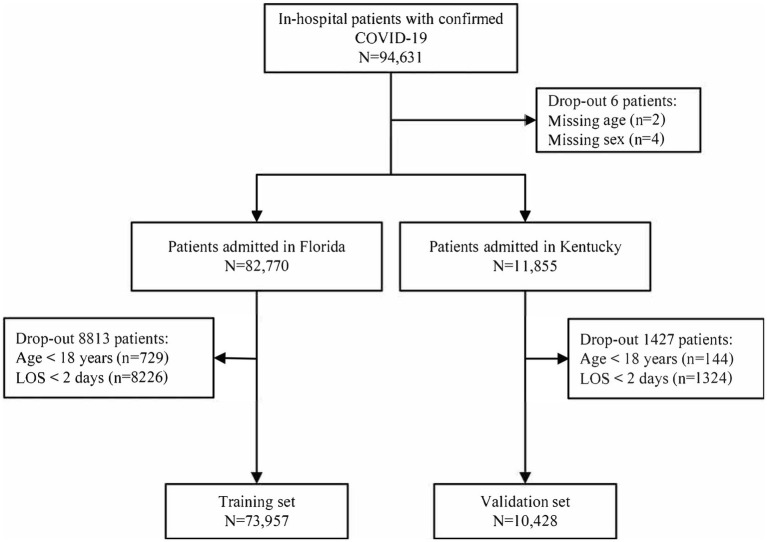
Flow chart of study participants in the training and validation sets.

A total of 94,631 patients who were hospitalized with COVID-19 underwent screening, resulting in the inclusion of 73,957 patients in the training and 10,428 patients in the validation set (Figure 1). The median age of the patients was 67 years (IQR, 54–78) and 47.27% were female patients. Among the patients, 44.02% belonged to the white ethnic group, whereas 54.74% belonged to non-white ethnic groups. The median LOS was 6 days (IQR, 4–11), and 39.45% patients had more than three comorbidities. Additionally, 8.13% of the patients received mechanical ventilation. The most prevalent comorbidities included uncomplicated hypertension (44.27%), obesity (27.83%), and diabetes with chronic complications (26.27%). In comparison to patients without mechanical ventilation, those receiving mechanical ventilation were more likely to be older, male, and have higher burden of comorbidities. Details regarding the baseline characteristics of the patients in the training and validation sets are presented in Table 1.

**Table 1:** Demographic and clinical characteristics for training and validation set of patients admitted to hospital with COVID-19.

Characteristics	Training set				Validation set			
	Overall (*n* = 73,957)	Not ventilated (*n* = 67,986)	Ventilated (*n* = 5,971)	*p* value	Overall (*n* = 10,428)	Not ventilated (*n* = 9,541)	Ventilated (*n* = 887)	*p* value
Age (median, IQR)	67 (55–78)	67 (54–79)	69 (59–77)	<0.001	63 (51–74)	63 (51–74)	67 (57–75)	<0.001
<60y	25,206 (34.08)	23,710 (34.87)	1,496 (25.05)		4,310 (41.33)	4,029 (42.23)	281 (31.68)	
60–69y	15,583 (21.07)	13,982 (20.57)	1,601 (26.81)		2,316 (22.21)	2089 (21.89)	227 (25.59)	
70–79y	16,470 (22.27)	14,627 (21.51)	1843 (30.87)		2,252 (21.60)	1989 (20.85)	263 (29.65)	
≥80y	16,698 (22.58)	15,667 (23.04)	1,031 (17.27)		1,550 (14.86)	1,434 (15.03)	116 (13.08)	
Female	35,239 (47.65)	32,880 (48.36)	2,359 (39.51)	<0.001	4,652 (44.61)	4,337 (45.46)	315 (35.51)	<0.001
Ethnicity				<0.001				<0.001
White	32,404 (43.81)	30,172 (44.38)	2,232 (37.38)		4,743 (45.48)	4,411 (46.23)	332 (37.43)	
Non-white	40,781 (55.14)	37,143 (54.63)	3,638 (60.93)		5,410 (51.88)	4,886 (51.21)	524 (59.08)	
Not recorded	772 (1.04)	671 (0.99)	101 (1.69)		275 (2.64)	244 (2.56)	31 (3.49)	
Number of comorbidities				<0.001				<0.001
≤3	44,114 (59.65)	41,769 (61.44)	2,345 (39.27)		6,984 (66.97)	6,570 (68.86)	414 (46.67)	
>3	29,843 (40.35)	26,217 (38.56)	3,626 (60.73)		3,444 (33.03)	2,971 (31.14)	473 (53.33)	
LOS (median, IQR)	6 (4–11)	6 (3–9)	17 (10–28)	<0.001	6 (4–10)	5 (3–9)	17 (10–27)	<0.001
AIDS	743 (1.00)	655 (0.96)	88 (1.47)	<0.001	40 (0.38)	36 (0.38)	4 (0.45)	0.956
Alcohol abuse	1,283 (1.73)	1,150 (1.69)	133 (2.23)	0.003	180 (1.73)	160 (1.68)	20 (2.25)	0.259
Deficiency anemias	15,433 (20.87)	13,367 (19.66)	2066 (34.60)	<0.001	1751 (16.79)	1,452 (15.22)	299 (33.71)	<0.001
Arthropathies	2,109 (2.85)	1935 (2.85)	174 (2.91)	0.794	209 (2.00)	190 (1.99)	19 (2.14)	0.856
Chronic blood loss anemia	223 (0.30)	189 (0.28)	34 (0.57)	<0.001	17 (0.16)	15 (0.16)	2 (0.23)	0.963
Leukemia	414 (0.56)	376 (0.55)	38 (0.64)	0.461	49 (0.47)	45 (0.47)	4 (0.45)	1
Lymphoma	478 (0.65)	410 (0.60)	68 (1.14)	<0.001	57 (0.55)	48 (0.50)	9 (1.01)	0.082
Metastatic cancer	561 (0.76)	516 (0.76)	45 (0.75)	1	53 (0.51)	48 (0.50)	5 (0.56)	1
Solid tumor without metastasis, *in situ*	9 (0.01)	8 (0.01)	1 (0.02)	1	1 (0.01)	1 (0.01)	0 (0.00)	1
Solid tumor without metastasis, malignant	1,262 (1.71)	1,152 (1.69)	110 (1.84)	0.428	159 (1.52)	146 (1.53)	13 (1.47)	0.994
Cerebrovascular disease	2,616 (3.54)	2,344 (3.45)	272 (4.56)	<0.001	245 (2.35)	211 (2.21)	34 (3.83)	0.003
Congestive heart failure	10,693 (14.46)	9,305 (13.69)	1,388 (23.25)	<0.001	1,356 (13.00)	1,151 (12.06)	205 (23.11)	<0.001
Coagulopathy	7,023 (9.50)	5,967 (8.78)	1,056 (17.69)	<0.001	955 (9.16)	795 (8.33)	160 (18.04)	<0.001
Dementia	11,540 (15.60)	10,895 (16.03)	645 (10.80)	<0.001	784 (7.52)	737 (7.72)	47 (5.30)	0.011
Depression	7,458 (10.08)	6,915 (10.17)	543 (9.09)	0.009	654 (6.27)	606 (6.35)	48 (5.41)	0.302
Diabetes with chronic complications	19,403 (26.24)	16,832 (24.76)	2,571 (43.06)	<0.001	2,767 (26.53)	2,383 (24.98)	384 (43.29)	<0.001
Diabetes without chronic complications	10,050 (13.59)	9,422 (13.86)	628 (10.52)	<0.001	1,525 (14.62)	1,438 (15.07)	87 (9.81)	<0.001
Drug abuse	1,210 (1.64)	1,098 (1.62)	112 (1.88)	0.142	192 (1.84)	173 (1.81)	19 (2.14)	0.571
Hypertension, complicated	19,360 (26.18)	16,915 (24.88)	2,445 (40.95)	<0.001	2,406 (23.07)	2077 (21.77)	329 (37.09)	<0.001
Hypertension, uncomplicated	32,969 (44.58)	30,692 (45.14)	2,277 (38.13)	<0.001	4,391 (42.11)	4,079 (42.75)	312 (35.17)	<0.001
Liver disease, mild	3,317 (4.49)	2,950 (4.34)	367 (6.15)	<0.001	513 (4.92)	467 (4.89)	46 (5.19)	0.762
Liver disease, moderate to severe	393 (0.53)	326 (0.48)	67 (1.12)	<0.001	52 (0.50)	41 (0.43)	11 (1.24)	0.002
Chronic pulmonary disease	17,263 (23.34)	15,705 (23.10)	1,558 (26.09)	<0.001	2,285 (21.91)	2046 (21.44)	239 (26.94)	<0.001
Neurological disorders affecting movement	2049 (2.77)	1896 (2.79)	153 (2.56)	0.327	194 (1.86)	182 (1.91)	12 (1.35)	0.299
Neurological disorders unaffecting movement	6,144 (8.31)	5,225 (7.69)	919 (15.39)	<0.001	882 (8.46)	687 (7.20)	195 (21.98)	<0.001
Seizures and epilepsy	2,670 (3.61)	2,381 (3.50)	289 (4.84)	<0.001	266 (2.55)	233 (2.44)	33 (3.72)	0.028
Obesity	20,739 (28.04)	18,399 (27.06)	2,340 (39.19)	<0.001	2,747 (26.34)	2,447 (25.65)	300 (33.82)	<0.001
Paralysis	2,355 (3.18)	2080 (3.06)	275 (4.61)	<0.001	231 (2.22)	196 (2.05)	35 (3.95)	<0.001
Peripheral vascular disease	2,937 (3.97)	2,590 (3.81)	347 (5.81)	<0.001	336 (3.22)	292 (3.06)	44 (4.96)	0.003
Psychoses	3,322 (4.49)	3,078 (4.53)	244 (4.09)	0.122	340 (3.26)	320 (3.35)	20 (2.25)	0.096
Pulmonary circulation disease	1881 (2.54)	1,602 (2.36)	279 (4.67)	<0.001	232 (2.22)	193 (2.02)	39 (4.40)	<0.001
Renal failure, moderate	9,189 (12.42)	8,097 (11.91)	1,092 (18.29)	<0.001	1,072 (10.28)	941 (9.86)	131 (14.77)	<0.001
Renal failure, severe	4,064 (5.50)	3,386 (4.98)	678 (11.35)	<0.001	664 (6.37)	548 (5.74)	116 (13.08)	<0.001
Hypothyroidism	10,328 (13.96)	9,476 (13.94)	852 (14.27)	0.492	1,277 (12.25)	1,175 (12.32)	102 (11.50)	0.512
Other thyroid disorders	815 (1.10)	729 (1.07)	86 (1.44)	0.011	123 (1.18)	114 (1.19)	9 (1.01)	0.754
Peptic ulcer with bleeding	329 (0.44)	273 (0.40)	56 (0.94)	<0.001	111 (1.06)	89 (0.93)	22 (2.48)	<0.001
Valvular disease	3,151 (4.26)	2,811 (4.13)	340 (5.69)	<0.001	332 (3.18)	305 (3.20)	27 (3.04)	0.882
Weight loss	4,377 (5.92)	3,721 (5.47)	656 (10.99)	<0.001	752 (7.21)	639 (6.70)	113 (12.74)	<0.001

IQR, interquartile range; LOS, length of stay; AIDS, Acquired immune deficiency syndrome.

### Model construction and performance assessment

**Figure 2:**
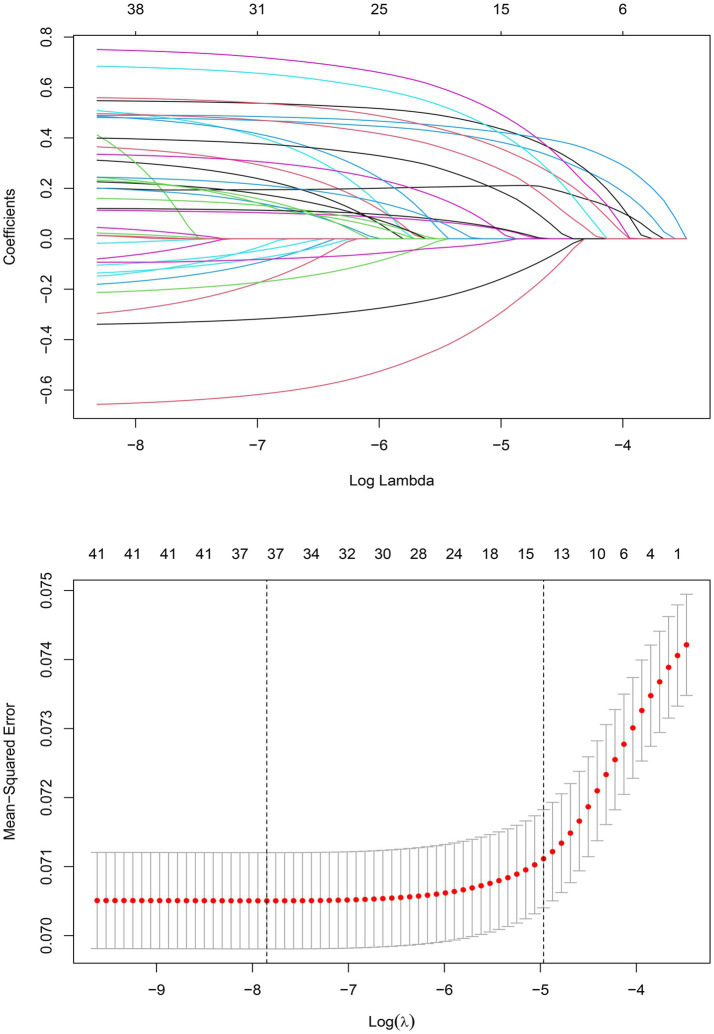
LASSO coefficient profiles of 40 candidate predictors **(A)** and 13 predictors selected using LASSO regression **(B)**.

**Figure 3:**
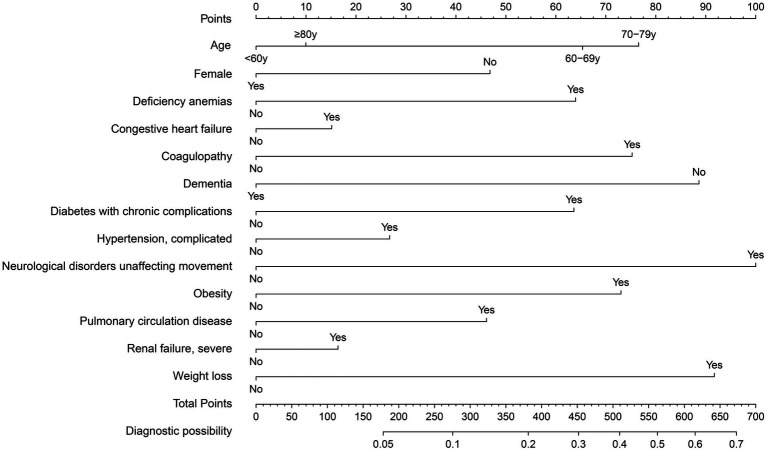
Nomogram for predicting mechanical ventilation requirement in patients with COVID-19.

The training set consisted of 73,957 patients, out of which 5,971 required mechanical ventilation. The LASSO method with 10-fold cross-validation was employed for feature selection among 40 candidate predictors, of which 13 predictor variables were identified and subsequently assessed by a multivariate logistic regression analysis (Figure 2). The variables associated with increased need for mechanical ventilation included older age, deficiency anemias, congestive heart failure, coagulopathy, diabetes with chronic complications, complicated hypertension, neurological disorders unaffecting movement, obesity, pulmonary circulation disease, severe renal failure, and weight loss (Table 2). Conversely, female and dementia were associated with decreased need for mechanical ventilation. It was notable that there was no significant difference in the need for mechanical ventilation between patients aged ≥80 years and those aged <60 years (OR, 1.08; 95% CI, 0.98–1.19). Subsequently, a nomogram incorporating age, sex, and the 11 aforementioned comorbidities was constructed to predict the need for mechanical ventilation by assigning a weighted score to each selected variable (Figure 3). The calculations of the total score was as follows: total score = 65 × (age, 60–69 years: yes = 1, no = 0) + 77 × (age, 70–79 years: yes = 1, no = 0) + 10 × (age, ≥80 years: yes = 1, no = 0) + 47 × (sex, female: yes = 0, no = 1) + 64 × (deficiency anemias: yes = 1, no = 0) + 15 × (congestive heart failure: yes = 1, no = 0) + 75 × (coagulopathy: yes = 1, no = 0) + 89× (dementia: yes = 0, no = 1) + 64 × (diabetes with chronic complications: yes = 1, no = 0) + 27 × (complicated hypertension: yes = 1, no = 0) + 100 × (neurological disorders unaffecting movement: yes = 1, no = 0) + 73 × (obesity: yes = 1, no = 0) + 46 × (pulmonary circulation disease: yes = 1, no = 0) + 16 × (severe renal failure: yes = 1, no = 0) + 92 × (weight loss: yes = 1, no = 0). The optimal cut-off point of the nomogram was determined to be 227, corresponding to a threshold probability of 0.071.

**Table 2:** Multivariable logistic regression of risk factors for mechanical ventilation support in patients with COVID-19 in the training set.

	*β*	OR	95% CI	*p* value
Intercept	−3.27			
Age (years)				<0.001
<60	Reference	Reference	Reference	
60–69	0.501	1.65	1.53–1.78	
70–79	0.587	1.8	1.67–1.94	
≥80	0.076	1.08	0.98–1.19	
Female	−0.36	0.7	0.66–0.74	<0.001
Deficiency anemias	0.49	1.63	1.53–1.74	<0.001
Congestive heart failure	0.12	1.12	1.03–1.22	0.007
Coagulopathy	0.58	1.78	1.65–1.92	<0.001
Dementia	−0.68	0.51	0.46–0.56	<0.001
Diabetes with chronic complications	0.49	1.63	1.53–1.73	<0.001
Hypertension, complicated	0.21	1.23	1.13–1.33	<0.001
Neurological disorders unaffecting movement	0.77	2.15	1.98–2.34	<0.001
Obesity	0.56	1.75	1.65–1.86	<0.001
Pulmonary circulation disease	0.35	1.42	1.24–1.64	<0.001
Renal failure, severe	0.13	1.13	1.02–1.26	0.016
Weight loss	0.7	2.02	1.84–2.22	<0.001

OR, odds ratio; CI, confidence interval.

**Figure 4:**
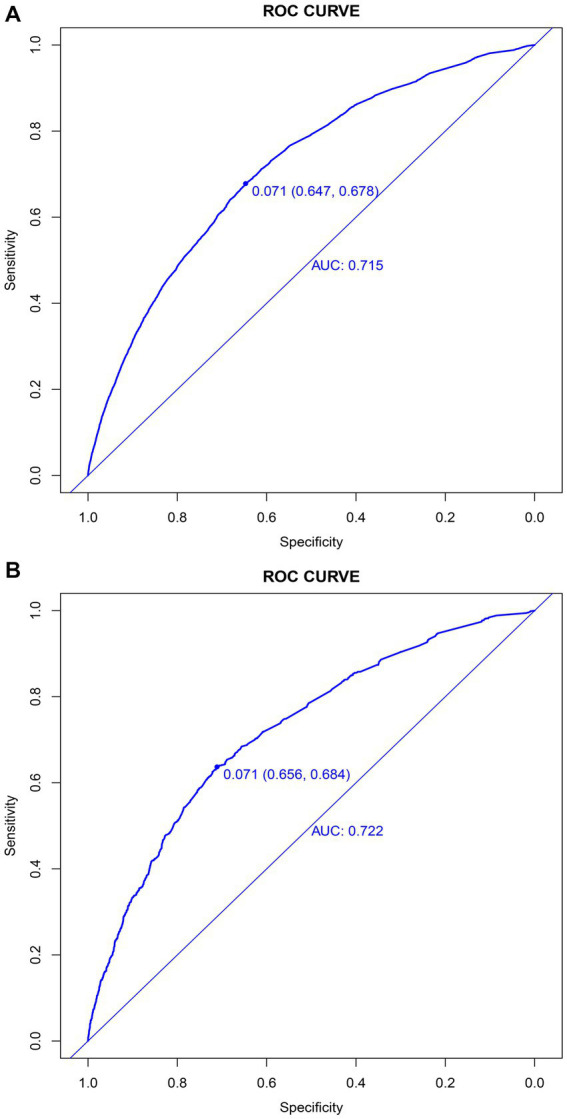
Discrimination of the nomogram for predicting mechanical ventilation requirement in patients with COVID-19. Receiver operator characteristic curves of the nomogram in the training **(A)** and validation sets **(B)**.

**Figure 5:**
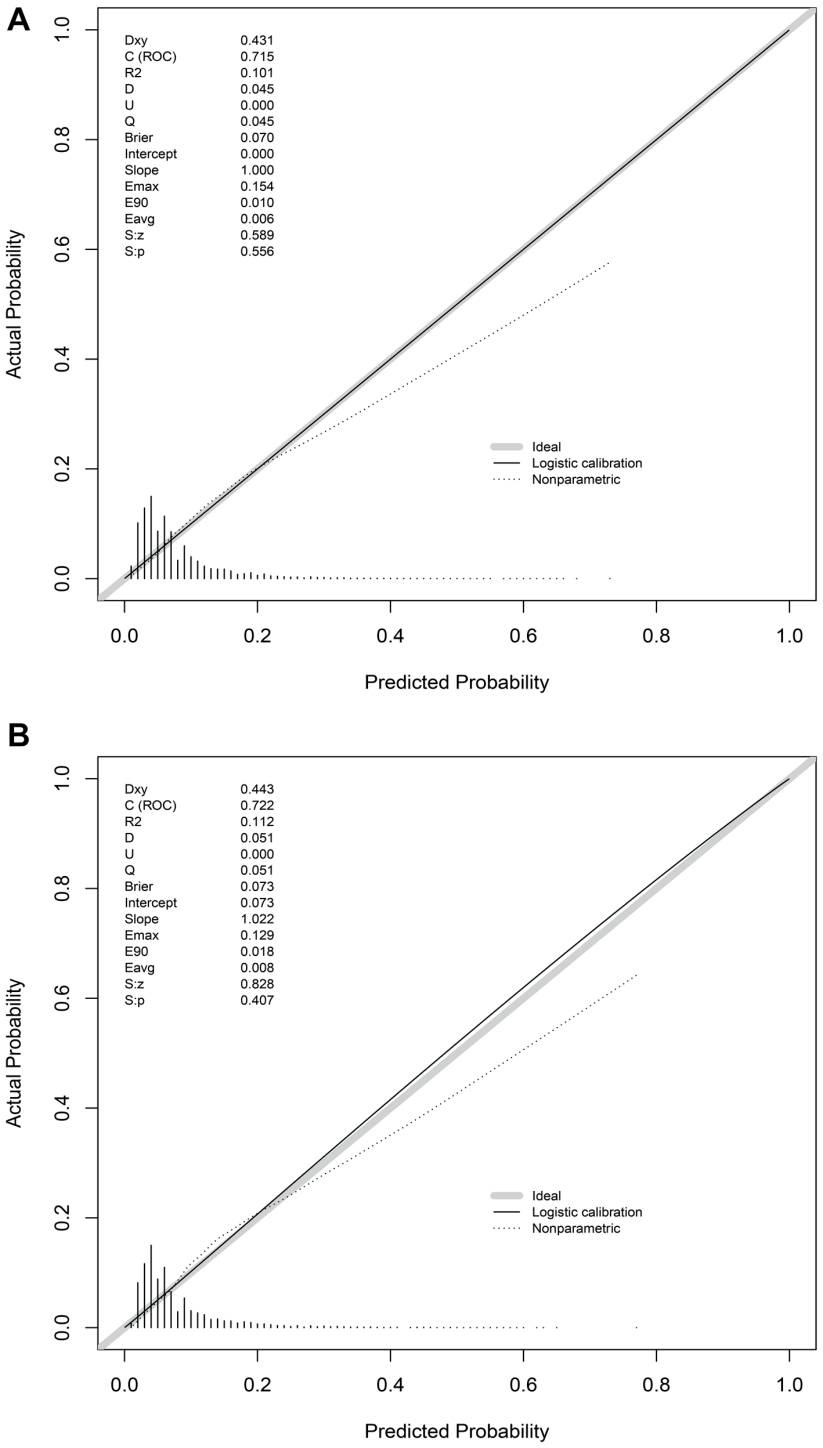
Calibration of the nomogram for predicting mechanical ventilation requirement in patients with COVID-19. Calibration curves of the nomogram in the training **(A)** and validation sets **(B)**.

**Figure 6:**
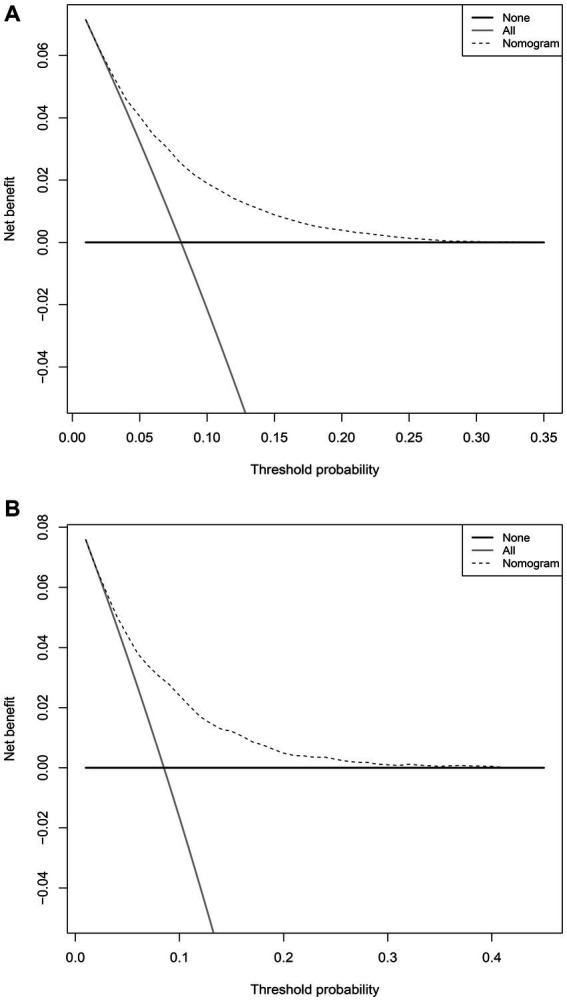
Decision curve analyses depicting the net benefits of the nomogram. The decision curve analyses of the nomogram in the training **(A)** and validation sets **(B)**.

In the training set, the nomogram exhibited a discriminatory performance for distinguishing patients who required mechanical ventilation from those who did not, with an area under the curve (AUC) of 0.715 (95% CI, 0.709–0.722). The cut-off value of 0.071 provided maximal discrimination, with a specificity of 0.647 and a sensitivity of 0.678 (Figure 4A). Furthermore, the calibration curve plotting the actual probability against the predicted probability demonstrated good calibration (Brier score = 0.070, slope = 1, intercept = 0) (Figure 5A). The DCA demonstrated that the nomogram had a superior clinical net benefit with a threshold probability range of 2–34%, when compared to the strategies of “treat all” or “treat none” (Figure 6A).

### Validation of the nomogram

The validation set consisted 10,428 patients of whom 887 patients required mechanical ventilation. In this set, the nomogram displayed comparable discrimination ability with an AUC 0.722 (95% CI, 0.704–0.739) (Figure 4B). Using the cut-off value of 0.071 identified in the training set, the specificity and sensitivity in the validation set were 0.656 and 0.684, respectively (Figure 4B). The calibration curve also demonstrated good agreement in the validation set (Brier score = 0.073, slope = 1.022, intercept = 0.073) (Figure 5B). The DCA illustrated that the clinical net benefit of the nomogram was higher than default strategy of “treat all” or “treat none,” with a threshold probability range of 3–42% (Figure 6B).

### Sensitivity analysis

In the datasets including patients aged <18 years or with an LOS < 2 days, the prediction model exhibited similar discrimination performance in both the training set (AUC, 0.720; 95% CI, 0.714–0.727; specificity, 0.664; sensitivity 0.671) and the validation set (AUC, 0.730; 95% CI, 0.713–0.747; specificity, 0.674; sensitivity, 0.681). After the stratification of the datasets by ethnicity, better discrimination performance was observed in non-white ethnic group (AUC, 0.728; 95% CI, 0.719–0.737; specificity, 0.666; sensitivity, 0.689) compared with white ethnic group (AUC, 0.699; 95% CI, 0.688–0.710; specificity, 0.623; sensitivity, 0.664), in the training set. Comparable results were observed in the validation set, with a lower discrimination performance observed in the white ethnic group (AUC, 0.690; 95% CI, 0.660–0.719; specificity, 0.635; sensitivity, 0.666) than in the non-white ethnic group (AUC, 0.743; 95% CI, 0.720–0.765; specificity, 0.673; sensitivity, 0.697).

## Discussion

In this study, we developed and evaluated a risk stratification model for predicting the need for mechanical ventilation in a large cohort including 84,025 patients hospitalized with COVID-19. The present model incorporates age, sex, and 11 other comorbidities, demonstrates moderate discrimination, good calibration, and clinical utility. Sensitivity analyses support the robustness of the model across different settings.

Compared with the present prediction model constructed using a hybrid method combining LASSO regression and multivariable logistic regression, several existing models using machine learning techniques have shown a moderate to good discrimination ability (AUC, 0.65–0.94) (6, 21–24). However, these existing models were commonly developed by cohorts with small sample size or the patients already admitted to the intensive care units, leading to an optimistic estimate of model performance or limiting their application in generally hospitalized patients. Our present prediction model was more interpretable and easier bedside to use, without need for an application or a website that hosts the calculator.

Age has consistently been identified as a strong predictor of adverse outcomes in patients with COVID-19 (25). Our study observed an increasing trend in risk of mechanical ventilation with age was observed in the current study, except for those aged ≥80 years. A study conducted in Japan found that patients aged ≥75 years had a lower rate of requirement for mechanical ventilation support compared with those aged 65–74 years (26). Similarly, another study using data from Korea also suggested that patients aged ≥80 years were less likely to receive mechanical ventilation (27). Considering the higher proportions of do-not-intubate (DNI) orders in older patients with COVID-19 (8), these results potentially reflect a clinical decision made in advance, rather than a lower risk of severe respiratory failure. Other factors such as medical resource availability, the potential harm and benefits of mechanical ventilation, and expected prognosis also contributed to the clinical decision-making for older patients (28, 29).

Comorbidity play a crucial role in predicting the prognosis of patients with COVID-19 (13). The predictive effect of comorbidities was usually presented in two forms in different risk stratification models: individual comorbidity unequally weighted and a count of comorbidities equally weighted (30, 31). In our cohort, we observed that neurological disorders unrelated to movement and weight loss exhibited the greatest ORs, which were significantly higher than those of the other comorbidities. In contrast with other comorbidities, dementia presented an oppositely predictive effect on mechanical ventilation (OR, 0.51; 95% CI, 0.46–0.56). Therefore, in our final prediction, we assigned weighted scores to individual comorbidity, rather than using an unweighted count of comorbidities. The association between dementia and decreased risk of mechanical ventilation may be explained by the fact that patients with dementia are more likely to have an advance care planning (ACP) or do-not-resuscitate (DNR) order, leading to a lower treatment intensity (32). After risk adjustment of ACP, dementia showed no significant effect on the likelihood of receiving mechanical ventilation (33).

The timely prediction of adverse outcomes of patients is of paramount important for effective allocation of the healthcare resources and prevention of clinical deterioration. However, prediction models which exhibit different performance in various subpopulations might potentially introduce unfairness in clinical decision-making and exaggerate health inequity (34). Underdiagnosis of mechanical ventilation requirement can result in delayed medical intervention, while overdiagnosis can lead to inappropriate aggressive treatment (35). The present model demonstrated similar performance between white ethnic group and non-white ethnic groups in terms of specificity and sensitivity, confirming that its application would not introduce unfairness in clinical decision-making.

One of the major strengths of our study was using a large, representative dataset to develop a model with a sufficient sample size, thereby reducing the risk of bias. Additionally, we also implemented LASSO regression for feature selection, a robust method that effectively mitigates multicollinearity within the model. Moreover, we performed sensitivity analyses to evaluate model’s discriminatory performance in different subpopulations and confirm its robustness.

However, this study has some limitations that should be acknowledged. First, the HCUP SID database did not provide details on DNR or DNI orders of patients, particularly those with greater age or dementia. This limitation might have influenced the analysis of truly requirement for mechanical ventilation in our study. Patient treatment preferences should be considered in the future studies to improve model performance. Second, the HCUP SID database did not record imaging and laboratory results, which were common predictor variables in other prediction models (8, 9). Consequently, it was impossible to compare their performance with our model in the present datasets. Third, all the patients included in this study were from US in 2020, which might limit the generalizability of the present model to a broader population. Moreover, the application of emerging vaccines and anti-viral agents, as well as the emergence of new COVID-19 variants, could exert influence on the risk of adverse outcomes (36–38). Hence, future validations using data from patients with COVID-19 from different pandemic periods and regions should be conducted to confirm the stability and generalizability of this prediction model.

## Conclusion

This study has presented a robust prediction model incorporating age, sex, and a set of comorbidities to assess the risk of receiving mechanical ventilation in hospitalized patients with COVID-19. Good performance of this risk stratification model was observed in discrimination, calibration, and clinical utility. The application of this model, incorporating predictor variables readily available at hospital admission, can facilitate early identification of the patients with a high-risk for mechanical ventilation, and assist front-line clinicians to optimize patient management and resource allocation during periods with a surge in infections and a limited supply of mechanical ventilators.

## Data availability statement

Publicly available datasets were analyzed in this study. This data can be found at: www.hcup-us.ahrq.gov.

## Ethics statement

The studies involving human participants were reviewed and approved by the Ethics Committee of the Naval Medical University. Written informed consent for participation was not required for this study in accordance with the national legislation and the institutional requirements.

## Author contributions

YZ, Y-JZ, and D-JZ contributed to the study design, data acquisition, statistical analysis, and manuscript preparation. B-YY and T-TL contributed to the study design and model development. L-YW contributed to the data acquisition and manuscript preparation. L-LZ contributed to the study conception, design, data interpretation, manuscript editing, and funding acquisition. All authors the read and approved the final manuscript.

## Funding

This work was supported by a grant from the National Science Foundation of China (no. 72174204) and Military Key Disciplines Construction Project (no. 03).

## Conflict of interest

The authors declare that the research was conducted in the absence of any commercial or financial relationships that could be construed as a potential conflict of interest.

## Publisher’s note

All claims expressed in this article are solely those of the authors and do not necessarily represent those of their affiliated organizations, or those of the publisher, the editors and the reviewers. Any product that may be evaluated in this article, or claim that may be made by its manufacturer, is not guaranteed or endorsed by the publisher.
